# Correction: Economic policy uncertainty, intra-industry trade, and China’s mechanical and electrical product exports

**DOI:** 10.1371/journal.pone.0305104

**Published:** 2024-06-04

**Authors:** Dajun Liu, Xiugang Zhu, Huiru Yu

The first two authors, Dajun Liu and Xiugang Zhu, should be noted as contributing equally and co-first authors of this work.
