# Expression of Concern: Economic policy uncertainty, intra-industry trade, and China’s mechanical and electrical product exports

**DOI:** 10.1371/journal.pone.0349909

**Published:** 2026-05-28

**Authors:** The PLOS One Editors

After this article [1,2] was published, the *PLOS One* Editors identified concerns about the article’s peer review and compliance with the PLOS Authorship policy. We regret that the issues were not addressed prior to the article’s publication.

In light of the cumulative issues, the *PLOS One* Editors issue this Expression of Concern. Readers are advised to interpret the article [1] with caution.
