# Supplementary material for: Economic policy uncertainty, intra-industry trade, and China’s mechanical and electrical product exports
Source: PLoS One. 2024 Jan 18;19(1):e0290866. doi: 10.1371/journal.pone.0290866 (PMC10795980; doi:10.1371/journal.pone.0290866)
Supplement: Attachment — Submitted filename: Review Report Plos One_PONE-D-22-06611.pdf [file pone.0290866.s001.pdf]

Review Report

Paper Title: Economic Policy Uncertainty, Intra-industry Trade and China's Mechanical and Electrical Product Exports

The authors have attempted to test the effect of economic policy uncertainty (EPU) and domestic intra-industry trade on China's mechanical and electrical product exports to 23 trading partners. The overall work is good. However, I feel that there is still some room to improve the paper before it can be published. My suggestions are as follows:

Abstract:

1. The abstract is well written with detailed findings. However, I recommend that the authors incorporate the importance of this work (why the author feels that this work is important) in a sentence in the beginning of the abstract.
2. Please try to stick to the same tense throughout the texts in the abstract. For instance, "this paper uses Using Chinese customs data on HS 8-digit products over the period of 2000–2013, this paper first investigated the impact of both foreign economic policy uncertainty (EPU) and domestic intra-industry trade on China's mechanical and electrical product exports to 23 trading partners and applies the methods of pooled OLS regressions to do an empirical study". Please use "investigates" instead of the past form to keep coherence before and after the "and" clause.
3. Also fix the error in the sentence "This paper uses Using Chinese customs data on HS 8-digit". It should be uses. And write the full form of HS please.

Introduction

1. Please clarify the concepts like" Markowitz portfolio strategies" and "real option theory" by writing a few more sentences. It will help to create a wider readership for this article.
2. Overall the introduction section is poorly written and needs sufficient modification. The authors must discuss the EPU concept extensively and link why policy uncertainties matter in the mechanical and electrical sectors the most. Try to discuss the effects of EPU on these sectors as well as other sectors and incorporate references from recent literature. This is important as the EPU plays a good role in the context of this paper.
3. Please give an overview of China's export sector incorporating the details and percentages of the total exports from different sectors. A graphical representation is highly recommended.
4. Why China? Why not other nations? Please clarify.
5. In the intro, please try to establish a robust link between the core of the problem and how you can address the issues empirically. At this moment it is missing.
6. The novelty statement of this paper is missing. Please highlight how the paper is about to enrich the current literature in a clearer fashion. A point-by-point presentation can enrich this section.
7. Include a different paragraph as follows: The remaining of the paper is designed as follows: Section 2 delineates the Literature Review……..

Literature review

1. Please try to use past tense in the literature review section. Write Author X documented instead of Author X document.
2. Incorporate a separate paragraph and write the overall gaps in the literature.

Methods

1. In section 3.2.2, the reference year for the EPU index is missing. In section 3.2.4., the sources of the data are missing.

Empirical Analysis and Conclusion

1. The results and discussion parts are well written. Include a note under all tables to denote what does the '***' stand for
2. The author should show that his findings are replicable and comparable with others. When discussing the outcomes, the author should cite from the recent literature to show that his findings are comparable. Please try to incorporate the recent progress in the literature in your discussion section.

Conclusion

1. Please include the limitations of the present study and also suggest the direction for any future studies.
