# Supplementary material for: Economic policy uncertainty, intra-industry trade, and China’s mechanical and electrical product exports
Source: PLoS One. 2024 Jan 18;19(1):e0290866. doi: 10.1371/journal.pone.0290866 (PMC10795980; doi:10.1371/journal.pone.0290866)
Supplement: Attachment — Submitted filename: PONE-D-22-06611R1.pdf [file pone.0290866.s003.pdf]

Review Report

Paper Title: Economic Policy Uncertainty, Intra-industry Trade and China's Mechanical and Electrical Product Exports

The authors have attempted to address the comments raised by me in the first round of the revision. However, some responses addressed by the authors are not satisfactory to accept the paper. The following points need to be addressed:

1. In the abstract, the authors have added two lines in the beginning. However, these do not justify why the work is important and it requires attention from the wider scholarly community. I recommend that the authors incorporate the importance of this work (why the author feels that this work is important).
2. "The study of this paper indicates that in the context of increasing uncertainty, our findings could have far-reaching policy implications for China to build a new development pattern of domestic and international dual circulation". Please write "The results of this paper indicate that …….
3. There are many scattered typos visible in the naked eye. Still the authors have failed to address them. This indicates lack of professionalism. On page 1, line 30, I see "0." attached to the citation (i.e., Handley, 2014[0.]). Why is that? Similar cases are also seen in lines 36, 52 …. Section 2.1, 2.2, 22.3 in the LR as well. I see them all through the manuscript. Please remove them all.
4. On page 1, line 41, it should be China's. Please read full manuscript with attention and care to fix all these typos. It is not possible to point out all different typos, given that it is your responsibility to identify and fix them.
5. Use a different color in figure 1, exclusively for the dotted line. Its not very clear at this point.
6. In the footnote 1, on page 2, what is 82-91?? Please clarify.
7. Line 51 is incorrectly written. Please fix the grammatical errors.
8. There are so too much smaller paragraphs in the intro. Merge them and produce reasonably sized paragraphs.
9. The novelty section should be written in a concrete structure. Currently, it is scattered. Place the novelty section in the end of the introduction. Elaborate the novelties within a point-by-point framework (i.e., firstly, secondly….).

10. I do not understand what the authors intend to say in line 64, “As shown in the figure 1……..”. It seems to be falling apart.
11. In the intro, please try to establish a robust link between the core of the problem and how you can address the issues empirically. At this moment it is missing. This comment was given in the initial round. Please take care of this comment. I did not ask the authors to provide justification about why they used the specific empirical model. Rather, I asked them to discuss the core problem based on which this paper has been written and the ways to tackle the core problem.
12. Start line 89 as, “The remaining of the manuscript is structured as follows:……”
13. Incorporate a separate paragraph in the end of the LR section and write the overall gaps in the literature.
14. There is no discussion on the theoretical mechanism and no discussion about figure 2
15. In table 1, highlight and bold the key points (such as explained variable, key explanatory variables, control variable)
16. On page 10, line 342, Kee and Tang, 20160, it should be 2016.
17. The authors have incorporated table notes under each of them. However, there are many visible spelling mistakes that the authors did not care to fix. They just copied and pasted same lines again and again. This is unprofessional and shameful. This is a revised version and the authors should put extra care in fixing the issues.
18. The English language needs substantial work and I recommend that the authors visit a professional proofreader to get the job done.
19. Please include the limitations of the present study and the direction for any future studies in the end of the conclusion. NOT in the middle.
