# Supplementary material for: Economic policy uncertainty, intra-industry trade, and China’s mechanical and electrical product exports
Source: PLoS One. 2024 Jan 18;19(1):e0290866. doi: 10.1371/journal.pone.0290866 (PMC10795980; doi:10.1371/journal.pone.0290866)
Supplement: Attachment — Submitted filename: Response to Reviewer Report.docx [file pone.0290866.s002.docx]

**Response to Reviewer Report on PONE-D-22-06611**

“Economic Policy Uncertainty, Intra-industry Trade, and China’s Mechanical and Electrical Product Exports”

We would like to thank you for your valuable comments and suggestions. Following these comments, we have thoroughly revised the manuscript. We believe that the quality of our manuscript has been greatly improved. The detailed responses are shown below. First, your comments and suggestions are errors in grammar and text that we have modified. Secondly, in the abstract and introduction part of your comments and suggestions are not rich and detailed enough, we have added the main conclusions of the article and the empirical test content, expanded the content of the theoretical part. Finally, we need to be improved in what your comments and suggestions are about the conclusions and policy suggestions, and we have added new content. Furthermore, in our resubmitted draft, the revised section is highlighted in blue.

**Response to Reviewer**

***The purpose of this paper is to empirically investigate the impact of economic policy uncertainty and domestic intra-industry trade on China's exports of electromechanical products to its 23 trading partners. Then the role of intra-industry trade on the regulation of EPU and export is further discussed. The authors point out that EPU has a significant inhibitory effect on the export of electromechanical products; instead, internal industrial trade can both significantly promote the export and alleviate the inhibitory effect of EPU. Moreover, due to the differences in trade patterns, EPU significantly inhibits processing and exports, but it has no impact on ordinary exports. The above results proved to be robust by endogenous problems.***

***Overall, this paper finds that the relationship between trade policy and exports should be valued, and I will continue to extend my attention and provide some relevant suggestions below.***

Thank you for the insightful comments. We have substantially revised our manuscript. Please see the revised paper and our responses below for more details.

**Comment 1:**

1. **Abstract**

***(1)The abstract is well written with detailed findings. However, I recommend that the authors incorporate the importance of this work (why the author feels that this work is important) in a sentence in the beginning of the abstract.***

**Response:** We greatly appreciate this comment.We have added sentences at the beginning of the abstract that illustrate the importance of this work.

“Nowadays,the world is undergoing profound changes unseen in a century. Economic policy uncertainties have had an important impact on trade and sustainable economic development.”

***(2)Temporal inconsistency problem***

***Please try to stick to the same tense throughout the texts in the abstract. For instance, “this paper uses Using Chinese customs data on HS 8-digit products over the period of 2000–2013, this paper first investigated the impact of both foreign economic policy uncertainty (EPU) and domestic intra-industry trade on China’s mechanical and electrical product exports to 23 trading partners and applies the methods of pooled OLS regressions to do an empirical study”. Please use “investigates” instead of the past form to keep coherence before and after the “and” clause.***

**Response:** Thank you for your suggestion, we have adjusted the tense in the article to unify into the present tense.

***(3)The HS form is incomplete***

***Also fix the error in the sentence “This paper uses Using Chinese customs data on HS 8-digit”. It should be uses. And write the full form of HS please.***

**Response:**Thanks for your suggestions.We have modified the statement correctly in the new revision, adding that the products starting with 82-91 in HS are electromechanical products (section 1,page 3).

***2.Introduction***

***(1) Interpretation theory***

***Please clarify the concepts like” Markowitz portfolio strategies” and “real option theory” by writing a few more sentences. It will help to create a wider readership for this article.***

**Response:**On the basis of your suggestions,We have added a detailed interpretation of the "Markowitz portfolio strategies "and" real option theory " two theories, so that readers can better understand the content of the article (section 1,page1-2).

“Real options theory is a modern theory on how to make decisions regarding investments when the future is uncertain. In the context of uncertainty, investors have two options: immediate investment and delayed investment in an investment decision. When the uncertainty risk is large, the immediate investment can obtain current investment profits but lose future possible profits, may also bear losses; delayed investment waits for the uncertainty to reduce the current investment profits but have the opportunity to obtain future possible profits, another possibility is that the uncertainty is further expanded, so the loss outweighs the gain.”

“Markowitz portfolio strategies is a Markowitz portfolio choice theory proposed in 1952, which holds that investors' investment desire is to pursue high expected returns and avoid risk as much as possible. That is, on the basis of the established risk level, how to make the possible expected return of securities great, or to obtain the established expected return, how to make the risk minimal.”

(2) ***Overall the introduction section is poorly written and needs sufficient modification. The authors must discuss the EPU concept extensively and link why policy uncertainties matter in the mechanical and electrical sectors the most. Try to discuss the effects of EPU on these sectors as well as other sectors and incorporate references from recent literature. This is important as the EPU plays a good role in the context of this paper.***

**Response:**We concur with your comments. We added more reasons about why the impact of choosing EPU has on electromechanical products, and later introduced new literature to illustrate the impact of EPU on other departments. Please see the revised paper for more details.

***(3)Increase export graphics***

***Please give an overview of China’s export sector incorporating the details and percentages of the total exports from different sectors. A graphical representation is highly recommended.***

**Response:**We greatly appreciate your feedback.We have collected the data on the export volume of mechanical and electrical products from 2000 to 2020, and made a chart on the data on the total export volume of mechanical and electrical products to explain the significant ratio of the export volume of mechanical and electrical products and the reasons for selecting this index (section1 ,page 3,figure 1).More details are shown below.

(%)

| Year | 比例 |
| --- | --- |
| 2000 | 42.2599 |
| 2001 | 44.6401 |
| 2002 | 48.2383 |
| 2003 | 51.9037 |
| 2004 | 54.5013 |
| 2005 | 56.0046 |
| 2006 | 56.6991 |
| 2007 | 57.4702 |
| 2008 | 57.5197 |
| 2009 | 59.3464 |
| 2010 | 59.1622 |
| 2011 | 57.185 |
| 2012 | 57.5648 |
| 2013 | 57.2503 |
| 2014 | 55.9604 |
| 2015 | 57.6527 |
| 2016 | 57.6391 |
| 2017 | 58.3855 |
| 2018 | 58.7254 |
| 2019 | 58.3703 |
| 2020 | 59.4835 |

Figure 1 The proportion of the export volume of mechanical and electrical products

***(4)Why China? Why not other nations? Please clarify.***

**Response:**Thank you for your comments,We chose China because China is the world's largest trading country and is representative (section 1,page 2).

***(5)In the intro, please try to establish a robust link between the core of the problem and how you can address the issues empirically. At this moment it is missing.***

**Response:**Thank you for your comments.Listen to your suggestions, we added content about the test of the robustness of the conclusion in the introduction. We first conducted OLS regression analysis on the hypothesis content, and then tested the robustness of the strength of the results, which proved that the conclusion is robust by solving the existing endogenous problems.We have added a new section to introduce this part in the revised version (section 1,page 3).

“To make the empirical results more credible, we addressed the endogeneity problem in three aspects. Firstly,We considered the missing variable deviation, and further considered the product country fixed effect according to the baseline regression.Secondly,we focused on the measurement error deviation, which mainly includes the measurement error of the EPU and the industry trade index. Finally, we took the reverse causality relationship between EPU and electromechanical product exports and exports into consideration, and the weighted arithmetic average method was used to recalate the EPU index. After considering the three endogenicity problems the conclusions remain robust.”

***(6)The novelty statement of this paper is missing. Please highlight how the paper is about to enrich the current literature in a clearer fashion. A point-by-point presentation can enrich this section.***

**Response:** Thanks for your suggestions.Based on the suggestions you gave (section 1,page 2), we have added the following content:

“Although there are many literatures have done a lot of research on the relationship between EPU and trade (e.g. Andrew,2019[5],Gervais,2018[6]), they do not consider the correlation between the characteristics of intra-industry trade and EPU. The impact of intra-industry trade may be more sensitive to EPU. At the same time, the existing literature less focuses on the impact of a certain industry, which ignoring the differences in the impact of uncertainty on the trade of different industries. Using Chinese customs data on HS 8-digit products over the period of 2000–2013, this paper first investigates the impact of both foreign economic policy uncertainty (EPU) and domestic intra-industry trade on China’s mechanical and electrical product exports to 23 trading partners and applies the methods of pooled OLS regressions to do an empirical study. We find that EPU has a significant inhibition effect on mechanical and electrical product exports; conversely, intra-industry trade can both significantly promote exports and alleviate the inhibition effect of EPU. In addition, the export impact of EPU varied with different trade patterns. It can significantly inhibit the processing exports, while it has no effect on ordinary exports.”

***3.Literature review***

***(1)Please try to use past tense in the literature review section. Write Author X documented instead of Author X document.***

**Response:**Thank you for pointing the above out.The issue is modified in the new revision.

***(2)Incorporate a separate paragraph and write the overall gaps in the literature.***

**Response:**Thank you for your comments.The existing literature does not consider the correlation between the characteristics of intra-industry trade and uncertainty, and the intra-industry trade may be more sensitive to the impact of uncertainty. At the same time, the existing literature less focuses on the impact of a certain industry, thus ignoring the differences in the impact of uncertainty on the trade of different industries (section 1,page 2).

.

***4.Methods***

***(1)In section 3.2.2, the reference year for the EPU index is missing. In section 3.2.4., the sources of the data are missing.***

**Response:**We greatly appreciate your feedback. We have added the reference year of the EPU index in the new revision section 3.2.2, completing the missing data sources in 3.2.4.

***5.Empirical Analysis and Conclusion***

***(1)The results and discussion parts are well written. Include a note under all tables to denote what does the ‘***’ stand for***

**Response:**Thank you for your comments. We have added table notes below all the tables in the article.

***(2)The author should show that his findings are replicable and comparable with others. When discussing the outcomes, the author should cite from the recent literature to show that his findings are comparable. Please try to incorporate the recent progress in the literature in your discussion section.***

**Response:**Thank you for your comments.In the Hypothesis 1 proof section (section 4.1, page 9), we cite two articles on the connection between EPU and trade to show that the conclusions obtained in this paper are comparable, consistent with the conclusion that EPU has an inhibitory impact on manufacturing export appreciation and agricultural imports. Please see the revised paper for more details.

6. ***Conclusion***
(1) ***Please include the limitations of the present study and also suggest the direction for any future studies.***

**Response:**Thank you for your comments.As with other papers, this paper has limitations. On the one hand, due to the limitation of data availability, we failed to directly test the impact of the recent trade friction. On the other hand, we did not directly identify the theoretical mechanism contained in the regression results (section 5,page 14).

**Comment 2:**

(1) ***The English language needs more work. There are many grammatical and typo mistakes in this manuscript. The paper needs to be edited by a native English speaker.***

**Response:**We greatly appreciate this comment.Grammatical errors and typos occurring in the article have been corrected in the new revision.

(2) ***The major defect of this study is the debate or argument is not clearly stated in the introduction session. Hence, the contribution is weak in this manuscript. The authors have presented a lot of data and figures in the introduction section. I would suggest the authors enhance your theoretical discussion and arrive at your theoretical argument.***

**Response:** On the basis of your suggestions, we further investigate the impact of TPU decline on pure exporters. We add some main conclusions to the introduction part, in detail how we deal with the endogenous problem, explain the contribution of this paper from both theoretical and empirical aspects, and finally add a section description content to explain the overall context of this article (section 1,page 3) .The additional road sections are included as follows:

“To make the empirical results more credible, we addressed the endogeneity problem in three aspects. Firstly,We considered the missing variable deviation, and further considered the product country fixed effect according to the baseline regression.Secondly,we focused on the measurement error deviation, which mainly includes the measurement error of the EPU and the industry trade index. Finally, we took the reverse causality relationship between EPU and electromechanical product exports and exports into consideration, and the weighted arithmetic average method was used to recalate the EPU index. After considering the three endogenicity problems the conclusions remain robust.”

(3) ***The motivations behind the selection of Economic Policy Uncertainty, Intra-industry Trade, and China’s Mechanical and Electrical Product Exports countries are not clear.***

**Response:** We concur with your comments.We added a research motivation in the beginning to illustrate the great impact of trade within the industry on uncertainty from the perspective of trade model.

(4) ***A graphical presentation of study hypothesis will make it more readable and attract the audience.***

**Response:** Thank you for your comments. We have added a research mechanism graph to show that intra-industry trade can reduce the inhibition of EPU on the export of mechanical and electrial products (section 2.3 ,page 6).More details are shown below.l

Figure 2 Mechanic diagram

(5) ***Introduction, literature review, and empirical results and discussion section should be critically evaluated by the authors. I recommend improving this section by critically analyzing the previous studies.***

**Response:**Thank you for your comments.We discuss the research of the existing literature, point out that it is rarely analyzed from the trade model, and then explain the innovation points of this article (section 1,page 2), adding the following content:

“Although there are many literatures have done a lot of research on the relationship between EPU and trade (e.g. Andrew,2019[5],Gervais,2018[6]), they do not consider the correlation between the characteristics of intra-industry trade and EPU. The impact of intra-industry trade may be more sensitive to EPU. At the same time, the existing literature less focuses on the impact of a certain industry, which ignoring the differences in the impact of uncertainty on the trade of different industries. Using Chinese customs data on HS 8-digit products over the period of 2000–2013, this paper first investigates the impact of both foreign economic policy uncertainty (EPU) and domestic intra-industry trade on China’s mechanical and electrical product exports to 23 trading partners and applies the methods of pooled OLS regressions to do an empirical study. We find that EPU has a significant inhibition effect on mechanical and electrical product exports; conversely, intra-industry trade can both significantly promote exports and alleviate the inhibition effect of EPU. In addition, the export impact of EPU varied with different trade patterns. It can significantly inhibit the processing exports, while it has no effect on ordinary exports.”

(6) ***Literature gap is missing at the end of the literature review section. In this regard, the authors should mention the drawbacks and limitations of the previous studies and also mention that how to address these issues solved in this study accordingly.***

**Response:**The existing literature does not consider the correlation between the characteristics of intra-industry trade and uncertainty, and the intra-industry trade may be more sensitive to the impact of uncertainty. At the same time, the existing literature focuses less on the impact of a certain industry, thus ignoring the differences in the impact of uncertainty on the trade of different industries. This paper focuses on the impact of uncertainty on the electromechanical industry trade, and makes up the gap in this part (section 1,page 2).

(7) ***The references of applied data are missing in the main text.***

**Response:**Thank you for your comments.We have added annotations of the data sources below the table in the new revision (section 3.2.4,page 9).

(8) ***More explanations and interpretations must be added for the results. In this regard, it is suggested to compare the results of the present research with some similar studies which is done before and more explanations and interpretations must be added for the Results, and discussion, which are not enough.***

**Response:**Thank you for your comments.In the Hypothesis 1 proof section (section 4.1, page 9), we cite two articles on the connection between EPU and trade to show that the conclusions obtained in this paper are comparable, consistent with the conclusion that EPU has an inhibitory impact on manufacturing export appreciation and agricultural imports. Please see the revised paper for more details.

(9) ***The authors should present the main findings in graphical form. It will increase the brevity and more readerships and attract more audience.***

**Response:**Thanks for your comments.Following your recommendations, we drew scatter plots of EPU increment and electromechanical increments to prove our conclusions (section 3.3, figure 3, page 8). More details are shown in the figure below.)

Figure 3 Scatter plot of EPU increment and electromechanical export volume increment

(10) ***The conclusion and policy recommendations are not well written. Authors should add more to this section, especially in the aspect of policy framing and implementation.***

**Response:** Thanks for your comments.The original content has now been replaced in the new revision (section 5,page 14)，adding the following content:

“The conclusion of this study also shows that processing trade, which is characterized by international procurement of intermediate products, cannot be a “talisman” for the export of mechanical and electrical products under an uncertain environment. Being at the low end of the value chain for a long time is more likely to become a “victim” under the fluctuation of the export environment. General trade is not sensitive to the uncertainty of economic policy ,because its production links are rooted in the domestic market. Therefore, on the one hand, we should actively make good use of the domestic intermediate products market for the domestic circulation, encourage processing trade enterprises to increase the domestic procurement rate of intermediate products to improve the domestic value chain and change the “order economy” into “demand economy”.This is of positive significance for stabilizing foreign trade under the increasing uncertainty of the international environment.”

(11) ***It would be appropriate to indicate future research directions at the end of the conclusion section just before references***

**Response:**Thanks for your comments.The existing literature has not consider the link between the characteristic of intra-industry trade and uncertainty.At the same time,the existing literature just focuses on the impact of a certain industry, and pay less attention to other industry fields. Future research can more focus on the impact of uncertainty on different industries trade.(section 5,page 14).
