# Supplementary material for: Economic policy uncertainty, intra-industry trade, and China’s mechanical and electrical product exports
Source: PLoS One. 2024 Jan 18;19(1):e0290866. doi: 10.1371/journal.pone.0290866 (PMC10795980; doi:10.1371/journal.pone.0290866)
Supplement: Attachment — Submitted filename: Response to Reviewer.docx [file pone.0290866.s004.docx]

**Response to Reviewer Report on PONE-D-22-06611**

“Economic Policy Uncertainty, Intra-industry Trade, and China’s Mechanical and Electrical Product Exports”

We would like to thank you for your valuable comments and suggestions. Following these comments, we have once again revised the manuscript. We believe that the quality of our manuscript has been greatly improved. The detailed responses are shown below. First, you point out that there are many grammatical errors in the paper, we have asked professionals to revise them; second, we have added instructions on the importance of the research content; and finally, we have made detailed modifications for your other suggestions. Furthermore, in our resubmitted draft, the revised section is highlighted in red.

**Response to Reviewer**

***The purpose of this paper is to empirically investigate the impact of economic policy uncertainty and domestic intra-industry trade on China's exports of electromechanical products to its 23 trading partners. Then the role of intra-industry trade on the regulation of EPU and export is further discussed. The authors point out that EPU has a significant inhibitory effect on the export of electromechanical products; instead, internal industrial trade can both significantly promote the export and alleviate the inhibitory effect of EPU. Moreover, due to the differences in trade patterns, EPU significantly inhibits processing and exports, but it has no impact on ordinary exports. The above results proved to be robust by endogenous problems.***

***Overall, this paper finds that the relationship between trade policy and exports should be valued, and I will continue to extend my attention and provide some relevant suggestions below.***

Thank you for the insightful comments. We have substantially revised our manuscript. Please see the revised paper and our responses below for more details.

**Comment:**

1. ***In the abstract, the authors have added two lines in the beginning. However, these do not justify why the work is important and it requires attention from the wider scholarly community. I recommend that the authors incorporate the importance of this work (why the author feels that this work is important).***

**Response:** We concur with your comments. We think that the extant related literature

rarely analyzes the nexus between uncertainty and trade on a specific industry, and our research focuses on the mechanical and electrical industry. Please see the revised paper for more details (section 1, page 1).

2. ***“The study of this paper indicates that in the context of increasing uncertainty, our findings could have far-reaching policy implications for China to build a new development pattern of domestic and international dual circulation”. Please write “The results of this paper indicate that ……***

**Response:** On the basis of your suggestions, our revised results are presented as follows (section 1, page 1):

“The results of this paper indicate that in the context of increasing uncertainty, our findings could have far-reaching policy implications for China to build a new development pattern of domestic and international dual circulation.”

3. ***There are many scattered typos visible in the naked eye. Still the authors have failed to address them. This indicates lack of professionalism. On page 1, line 30, I see “0.” attached to the citation (i.e., Handley, 20140.). Why is that? Similar cases are also seen in lines 36, 52 …. Section 2.1, 2.2, 22.3 in the LR as well. I see them all through the manuscript. Please remove them all.***

**Response:** Thank you for your comments. Listen to your suggestions, we rechecked the entire paper and revised. Please see the revised paper for more details.

4. ***On page 1, line 41, it should be China’s. Please read full manuscript with attention and care to fix all these typos. It is not possible to point out all different typos, given that it is your responsibility to identify and fix them.***

**Response:**Thanks for your suggestions. Based on the suggestions you gave, we have modified this spelling error (section 1, page 2).

5. ***Use a different color in figure 1, exclusively for the dotted line. Its not very clear at this point.***

**Response:** Thank you for your comments. We modified Fig 1, more details are shown below.

(%)

| Year | 比例 |
| --- | --- |
| 2000 | 42.2599 |
| 2001 | 44.6401 |
| 2002 | 48.2383 |
| 2003 | 51.9037 |
| 2004 | 54.5013 |
| 2005 | 56.0046 |
| 2006 | 56.6991 |
| 2007 | 57.4702 |
| 2008 | 57.5197 |
| 2009 | 59.3464 |
| 2010 | 59.1622 |
| 2011 | 57.185 |
| 2012 | 57.5648 |
| 2013 | 57.2503 |
| 2014 | 55.9604 |
| 2015 | 57.6527 |
| 2016 | 57.6391 |
| 2017 | 58.3855 |
| 2018 | 58.7254 |
| 2019 | 58.3703 |
| 2020 | 59.4835 |

Figure 1 The proportion of the export volume of mechanical and electrical products

6. ***In the footnote 1, on page 2, what is 82-91?? Please clarify.***

**Response:** Thank you for your comments. We have added the meaning of this number in this paper. More details are shown below (section 1, page 3).

“The mechanical and electrical products mentioned in the article refer to the products whose HS code range from 82 to 91 in the customs data.”

7. ***Line 51 is incorrectly written. Please fix the grammatical errors.***

**Response:** Thanks for your suggestions. We have fixed these grammatical errors, more details are shown below (section 1, page 2).

“Extensive literature on the relationship between EPU and trade (e.g., Andrew,2019 [6], Gervais, 2018 [7]) does not consider the correlation between the characteristics of intraindustry trade and EPU.”

8. ***There are so too much smaller paragraphs in the intro. Merge them and produce reasonably sized paragraphs.***

**Response:** Thank you for your suggestions. Listen to your suggestions, we have integrated the passages of this paper. More details are shown below (section 1, page 3).

9. ***The novelty section should be written in a concrete structure. Currently, it is scattered. Place the novelty section in the end of the introduction. Elaborate the novelties within a point-by-point framework (i.e., firstly, secondly….).***

**Response:** Thank you for your suggestions. Listen to your suggestions, we have made the modifications. More details are shown below (section 1, page 4).

“The marginal contribution of this paper is mainly reflected in three aspects. First, the nexus between uncertainty and exports is analyzed from the perspective of a specific industry. The extant related literature mainly analyzes the nexus between uncertainty and trade across different industries and focuses less on a specific industry. Second, we empirically test the moderation effect of intraindustry trade and trade patterns on the relationship between uncertainty and exports based on highly disaggregated trade data. Third, the conclusions have some crucial policy implications. Further involvement in intraindustry trade and diversification of trade patterns can help firms alleviate the impact of external shocks due to policy uncertainty.”

***10. I do not understand what the authors intend to say in line 64, “As shown in the figure 1……..”. It seems to be falling apart.***

**Response:** Thank you for your comments. We have reillustrated Fig 1. More details are shown below (section 1, page 2).

“ As shown in Fig. 1, the proportion of exports of electrical and mechanical products to total exports has been above 50% since 2003.”

***11. In the intro, please try to establish a robust link between the core of the problem and how you can address the issues empirically. At this moment it is missing. This comment was given in the initial round. Please take care of this comment. I did not ask the authors to provide justification about why they used the specific empirical model. Rather, I asked them to discuss the core problem based on which this paper has been written and the ways to tackle the core problem.***

**Response:** Thank you for your suggestions. Listen to your suggestions, we have added to this content in the introduction. More details are shown below (section 1, page 3).

“The core issue of this paper is to identify the impact of EPU on mechanical and electrical products and the moderating role of intraindustry trade. To make the empirical results more credible, we addressed the endogeneity problem in three aspects. First, the omitted variable generally causes an error term correlated with other explanatory variables, which leads to a violation of the exogenous assumption. To solve this problem, we further control the product-country fixed effects according to the baseline regression. Second, the presence of measurement error biases causes explanatory variables to be correlated with perturbation terms, resulting in an endogeneity problem, so we use the weighted arithmetic mean method to recalculate the EPU index. Finally, reverse causality also causes a potential endogeneity problem, and we lag the intraindustry trade index for one period as a proxy to address this problem. After alleviating the three endogeneity problems, the conclusions remain robust.”

12. ***Start line 89 as, “The remaining of the manuscript is structured as follows:……”***

**Response:** Thank you for your suggestions. Listen to your suggestions, we have made the modifications. More details are shown below (section 1, page 4).

“The remainder of the paper is structured as follows. We show the relevant literature and theoretical hypotheses in Section 2. Based on the related literature, we propose three theoretical hypotheses to be tested. We present the estimation strategy in Section 3, mainly by establishing the regression models. Section 4 shows the regression results, including the benchmark regression results and a series of robustness tests. Section 5 presents the conclusion and policy implications.”

13. ***Incorporate a separate paragraph in the end of the LR section and write the overall gaps in the literature.***

**Response:** Thank you for your suggestions. Listen to your suggestions, we have made relevant supplements. More details are shown below (section 1, page 2).

“Extensive literature on the relationship between EPU and trade (e.g., Andrew,2019 [6], Gervais, 2018 [7]) does not consider the correlation between the characteristics of intraindustry trade and EPU. The impact of intraindustry trade may be more sensitive to EPU. At the same time, the literature focuses less on the impact of a certain industry and ignores the differences in the impact of uncertainty on the trade of different industries. This paper focuses on the impact of EPU on exports of the mechanical and electrical products industry and conducts an empirical study using customs data. We find that EPU has a significant inhibition effect on mechanical and electrical product exports; conversely, intraindustry trade can both significantly promote exports and alleviate the inhibition effect of EPU. In addition, the export impact of EPU varied with different trade patterns. It can significantly inhibit processing exports, while it has no effect on ordinary exports.”

14. ***There is no discussion on the theoretical mechanism and no discussion about figure 2***

**Response:** On the basis of your suggestions, we have added an explanation of the theoretical mechanism and Fig 2. More details are shown below (section 1, page 6).

Figure 2 Theoretical Mechanism

“Fig. 2 is the mechanism diagram depicted in this paper, where H1 illustrates the inhibitory effect of EPU on the export of mechanical and electrical products, H2 indicates the promotion effect of intraindustry trade on the export of mechanical and electrical products, and H3 indicates that it can alleviate the inhibitory effect of EPU on the export of mechanical and electrical products.”

15. ***In table 1, highlight and bold the key points (such as explained variable, key explanatory variables, control variable)***

**Response:** On the basis of your suggestions, we have modified the tables in this paper. More details are shown below.

16. ***On page 10, line 342, Kee and Tang, 20160, it should be 2016.***

**Response:** On the basis of your suggestions, we have fixed this error in this paper. More details are shown below (section 1, page 12).

17. ***The authors have incorporated table notes under each of them. However, there are many visible spelling mistakes that the authors did not care to fix. They just copied and pasted same lines again and again. This is unprofessional and shameful. This is a revised version and the authors should put extra care in fixing the issues.***

**Response:** On the basis of your suggestions, we have modified the table notes in this paper. More details are shown below.

18. ***The English language needs substantial work and I recommend that the authors visit a professional proofreader to get the job done.***

**Response:** On the basis of your suggestions, we have invited professionals to proofread the paper.

19. ***Please include the limitations of the present study and the direction for any future studies in the end of the conclusion. NOT in the middle.***

**Response:** On the basis of your suggestions, we have supplemented the relevant content in the end of this paper. More details are shown below (section 1, page 14).

“The existing literature has not fully considered the link between the characteristics of intraindustry trade and policy uncertainty. Moreover, the existing literature has paid little attention to the impact of specific industry trade. Future research can discuss the impact of uncertainty on the trade of other industries.”
