# Supplementary material for: Economic policy uncertainty, intra-industry trade, and China’s mechanical and electrical product exports
Source: PLoS One. 2024 Jan 18;19(1):e0290866. doi: 10.1371/journal.pone.0290866 (PMC10795980; doi:10.1371/journal.pone.0290866)
Supplement: Attachment — Submitted filename: Response to Reviewer.docx [file pone.0290866.s005.docx]

**Response to Reviewer Report on PONE-D-22-06611**

“Economic Policy Uncertainty, Intra-industry Trade, and China’s Mechanical and Electrical Product Exports”

We would like to thank you for your valuable comments and suggestions. Following these comments, we have thoroughly revised the manuscript. We believe that the quality of our manuscript has been greatly improved. The detailed responses are shown below. First, your comments and suggestions are errors in grammar and text that we have modified. Secondly, in the abstract and introduction part of your comments and suggestions are not rich and detailed enough, we have added the main conclusions of the article and the empirical test content, expanded the content of the theoretical part. Finally, we need to be improved in what your comments and suggestions are about the conclusions and policy suggestions, and we have added new content. Furthermore, in our resubmitted draft, the revised section is highlighted in blue.

**Response to Reviewer**

***The purpose of this paper is to empirically investigate the impact of economic policy uncertainty and domestic intra-industry trade on China's exports of electromechanical products to its 23 trading partners. Then the role of intra-industry trade on the regulation of EPU and export is further discussed. The authors point out that EPU has a significant inhibitory effect on the export of electromechanical products; instead, internal industrial trade can both significantly promote the export and alleviate the inhibitory effect of EPU. Moreover, due to the differences in trade patterns, EPU significantly inhibits processing and exports, but it has no impact on ordinary exports. The above results proved to be robust by endogenous problems.***

***Overall, this paper finds that the relationship between trade policy and exports should be valued, and I will continue to extend my attention and provide some relevant suggestions below.***

Thank you for the insightful comments. We have substantially revised our manuscript. Please see the revised paper and our responses below for more details.

**Comment :**

（1）***Fix the following: change "intraindustry" to "intra-industry". This error is prevalent all throughout the manuscript from title to the conclusion section. Proofread the paper and fix all.***

**Response:**On the basis of your suggestions,I have changed the full paper of "intraindustry" to "intra-industry".Please see the revised paper for more details.

（2）***Add a motivation section in the introduction to discuss what motivates you to write this paper.***

**Response:**We concur with your comments.Below is the specific content of the changes provided.

“ Extensive literature on the relationship between EPU and trade (e.g., Andrew,2019 [6], Gervais, 2018 [7]) does not consider the correlation between the characteristics of intra-industry trade and EPU. The impact of intra-industry trade may be more sensitive to EPU. At the same time, the literature focuses less on the impact of a certain industry and ignores the differences in the impact of uncertainty on the trade of different industries. In this context, the paper focuses on the electromechanical products industry to address this research gap. This paper aims to clarify the impact mechanism of trade uncertainty on export trade within specific industries. On one hand, it will facilitate our understanding of the changing trends in foreign trade under the backdrop of uncertainty from a global value chain perspective. On the other hand, it will be beneficial in providing relevant policy insights for emerging market countries like China to address economic policy uncertainties.This paper focuses on the impact of EPU on exports of the mechanical and electrical products industry and conducts an empirical study using customs data. We find that EPU has a significant inhibition effect on mechanical and electrical product exports; conversely, intra-industry trade can both significantly promote exports and alleviate the inhibition effect of EPU. In addition, the export impact of EPU varied with different trade patterns. It can significantly inhibit processing exports, while it has no effect on ordinary exports.”

***（3）The theoretical framework is missing. Please add a theoretical framework section in the beginning of the methodology and discuss the theories of the relevant research questions and how they are associated with your research hypotheses. "Theoretical mechanism" does not convey the theoretical underpinnings.***

**Response:**We concur with your comments.Below is the specific content of the changes provided.

“The classic theories relevant to this paper are primarily the theory of monopolistic competition in international trade (Krugman 1980[32];Melitz 2003[33] ). This theory is mainly based on empirical facts from developed countries and explains the role of internal economies of scale and firm heterogeneity in international trade patterns. Subsequently, some scholars incorporated uncertainty into the analytical framework and extended the models of heterogeneous firm trade from various perspectives (Handley and Limão, 2022 [1] ,2017[5] ).The theoretical framework of this article primarily highlights the significant role of economic policy uncertainty in global trade, particularly when it comes to specific industries, where uncertainty can sharply increase. Within this theoretical framework, the article explores the relevant research questions, and the theoretical mechanism is illustrated in Fig. 2.”

***（4）Please make sure that all titles and sub-titles are written in sentence case format (i.e., section 4.1 is different). Or at least they should follow the same pattern.***

**Response:** Thank you for your comments.I have made sure that all titles and sub-titles in sentence case format.Please see the revised paper for more details.

***（5）Rename section 5 as conclusions and policy implications. You should have at least three paragraphs here. First, convey what you did and convey the main results. Second, you should have the policy implications in a point-by-point framework. And third, you should have the limitations and prospect for future studies.***

**Response:**Thank you for your comments.I have already renamed Section 5 as Conclusions and Policy Implications, and made the necessary modifications to the paragraph content as you requested.Please see the revised paper for more details.

***（6）PLOS authors have the option to publish the peer review history of their article (what does this mean?). If published, this will include your full peer review and any attached files.***

**Response:**Thank you for your comments.
